# Factors Associated with Uptake of Intermittent Preventive Treatment of Malaria in Pregnancy: A Cross-Sectional Study in Private Health Facilities in Tema Metropolis, Ghana

**DOI:** 10.1155/2019/9278432

**Published:** 2019-08-01

**Authors:** Selina Amankwah, Francis Anto

**Affiliations:** ^1^School of Public Health, University of Ghana, Legon, Ghana; ^2^La General Hospital, La, Accra, Ghana

## Abstract

**Introduction:**

Intermittent preventive treatment of malaria in pregnancy with sulfadoxine pyrimethamine (IPTp-SP) is effective in preventing the adverse consequences of malaria on birth outcomes.

**Methods:**

A cross-sectional survey was carried out among antenatal and postnatal women and midwives at private health facilities in Tema using the mixed method to investigate factors associated with uptake of IPTp-SP. Antenatal and postnatal women were consecutively enrolled and data on their sociodemographic characteristics and antenatal service utilization collected using a questionnaire and review of antenatal care (ANC) records. In-depth interviews involving attending midwives were conducted and data on ANC service delivery collected. The interviews were manually analyzed. Bivariate and multiple logistic regression analyses were done to determine factors associated with uptake of SP.

**Results:**

Of the 382 respondents, 178 (46.6%) took ≥ 3 doses of SP. Uptake was similar for those who had delivered and those yet to deliver (*χ*^2^ =2.94, p > 0.05). Ninety-seven of the 176 (55.1%) women who initiated antenatal visit during the first trimester received ≥ 3 doses of SP whilst 42.0% (76/181) of those who started during the second trimester received ≥ 3 doses (*χ*^2^ = 5.64, p = 0.02). Those who initiated ANC during the second trimester received more doses compared to those who started during the third trimester (*χ*^2^ = 4.43, p = 0.04). Respondents who attended ANC > 5 times increased their uptake by 83% compared to those who attended < 5 times (OR 0.2, 95% C.I 0.12-0.31). There was poor adherence to directly observed treatment and low knowledge of midwives on IPTp-SP protocol.

**Conclusion:**

Early initiation and regular visit to antenatal care centres promoted uptake of optimal doses of SP.

## 1. Background

Malaria is an important vector-borne disease caused by* Plasmodium *parasites. Transmission of the parasites is through the bites of infected female* Anopheles* mosquitoes. Though the disease is preventable and curable, there were 219 million cases in 2017 and 435000 deaths worldwide with children under five years of age and pregnant women being the most affected [1]. Malaria infection in pregnancy is of public health significance as it adversely affects the pregnant woman, her fetus, and the new born baby. The malaria parasite species most associated with poor pregnancy outcomes including low birth weight, spontaneous abortion, stillbirth, premature labour, and maternal illness in Africa is* Plasmodium falciparum *[2].

In Ghana, malaria is hyperendemic and still a serious public health problem for pregnant women and children under five years even though effective interventions have been put in place to protect these highly vulnerable populations over the past years. The disease accounts for 17.6% of OPD attendance, 13.7% of admissions, and 3.4% of maternal deaths [3].

For effective and efficient prevention and control of malaria in pregnancy, the WHO has made some recommendations. These include the use of long-lasting insecticidal nets (LLINs) in all areas with moderate to high malaria transmission in Africa, intermittent preventive treatment in pregnancy (IPTp) with sulfadoxine-pyrimethamine (SP), and prompt diagnosis and effective treatment of malaria infections using artemisinin-based combination treatment [2].

Intermittent Preventive Treatment in pregnancy with SP (IPTp-SP) is an effective strategy in preventing the adverse consequences of malaria on maternal and fetal outcomes. In 2012, WHO updated her recommendations and now requires that at least three doses of SP be given to all pregnant women at each scheduled antenatal care (ANC) visit starting as early as possible in the second trimester and given at one-month intervals [4].Thus, every pregnant woman in areas with moderate to high malaria transmission in Africa is expected to receive at least three doses of SP to prevent malaria. However, during the last few years, a declining effort to scale-up IPTp-SP in a number of high-burden countries in Africa has been observed even though attendance at antenatal clinic has remain high [5].

The Ghana National Malaria Control Program (NMCP) also updated her policy and now recommends a minimum of five doses of SP [6]. Ghana's goal of achieving 55% uptake of at least three doses of SP by pregnant women in 2015 was not realized as only 41.3% received three or more doses of SP during the period [7]. Recent studies in some parts of the Greater Accra region of Ghana have reported significant improvement in the uptake of three or more doses [8].

According to the NMCP of Ghana, most private health facilities report low uptake of SP in the Tema metropolis. Out of the 68,629 pregnant women who sought ANC services at private facilities in the Tema metropolis in 2015, only 11.1% received three doses of SP, 5.5% received four doses, and 2.2% received five doses [3]. The control program considers this low level of uptake of IPTp-SP among users of private health facilities as a serious challenge to achieving its targets. The purpose of the current study therefore was to identify factors associated with uptake of SP among pregnant women who received ANC services at selected private hospitals in the Tema metropolis of Ghana that could be exploited to address these challenges.

## 2. Materials and Methods

### 2.1. Study Area

The study was carried out in the Tema metropolis of the Greater Accra region of Ghana. Tema metropolis is located along the coast of the Gulf of Guinea about 30 kilometers east of Accra, the capital city of Ghana, and has a population of about 293,000. For health administrative purposes, the area has been divided into three submetropolis. These are Tema East, Tema West and Tema Central. The metropolis has a total of 87 health facilities providing antenatal and postnatal services with more than half of these facilities being privately owned [9].

### 2.2. Study Design

A cross-sectional study was conducted at the antenatal and postnatal units of selected private hospitals in the Tema metropolis of Ghana using a mixed method approach. Pregnant women in their last two months of pregnancy and nursing mothers who had delivered within the past six months and had attended private hospitals for antenatal care services throughout the most recent pregnancy were enrolled into the study. Data on demographic characteristics, knowledge on SP, number of antenatal care visits, and experiences with SP side effects were collected from the women using an interviewer administered questionnaire. The antenatal care cards of the women were also reviewed and data extracted. In-depth interviews were organized for midwives in-charge of the antenatal care units of the facilities using an interview guide. Data on knowledge on intermittent preventive treatment in pregnancy using SP, compliance with directly observed treatment, supply of logistics by programme implementers, and participation in training programmes organized by the programme implementers were collected.

### 2.3. Sample Size Estimation and Sampling

The sample size for the study was calculated using the formula: n = Z^2^p(1-p)/d^2^, by Naing, Winn, & Rusli [10], where n = the estimated sample size, Z = standard value for 95% confidence level = 1.96, p = estimated level of IPTp 3 uptake among pregnant women receiving antenatal care in private hospitals in Tema metropolis = 30% or 0.3 [9], and d = margin of error = 5% or 0.05. The minimum required sample size estimated was 323. For the qualitative arm of the study, one midwife from each hospital was recruited for participation in the study.

Four health facilities were randomly selected from a list of health facilities providing both antenatal and postnatal services in each of the three submetropolis. The selected facilities were Port Medical Centre, Lagoon clinic, and TMA maternity and Meridian clinic (from Tema East); Fiden Medical Centre, Sun City Medical Centre, Trinity Community Hospital and Speed Medical Center (from Tema West) and Rapha Medical Center, Mother of God Hospital, Bethel Hospital, and New Crystal Hospital (from Tema Central). The mothers were consecutively enrolled into the study from each selected facility after having been attended to by the care givers of the hospitals on the days of the survey. Enrolment into the study was done simultaneously at all the health facilities ensuring that at least 32 participants were enrolled from each hospital. The midwife in-charge of the ANC unit of each facility was contacted for the in-depth interviews.

### 2.4. Inclusion/Exclusion Criteria

Pregnant women in their last two months of pregnancy had been attending ANC at a private hospital, or postnatal mothers who delivered within six months prior to the study, attended private hospitals for ANC during pregnancy, and gave written informed consent.

### 2.5. Data Collection Procedure

Data on sociodemographic characteristics including age, educational level, number of children, occupation, and marital status were collected directly from the mothers onto a data collection form designed specifically for this study. Data on patient factors likely to influence uptake of IPTp-SP such as knowledge about SP, previous experience of SP side effects, and number of ANC visits during the most recent pregnancy were also collected from the mothers. Some of these data including number of ANC visits were confirmed using the ANC cards of the mothers and ANC registers of the units.

Also, data on ANC services including availability of SP at the ANC clinic and swallowing the drug under supervision were also collected directly from the mothers. For the purpose of accuracy, data on gestational age at first ANC visit, number of doses of SP taken before delivery or at the current gestational age, and the gestational age at which first dose of SP was taken, were extracted from the ANC record cards of the mothers. Where there was discrepancy between the verbal information given and that written on the ANC card, what was on the card was used. For those without antenatal cards data collected were based on the interviews and ANC registers.

The midwives in-charge of the ANC units were interviewed in their offices one-on-one using an interview guide with coded themes to direct the flow of interaction. Information on their knowledge on IPTp-SP, compliance to the DOT policy, support from programme implementers, and counselling of patients were collected from them. An audio recorder was used to record all interviews and one trained research assistant took notes of nonverbal expressions and other important points. The interviews lasted for 30-45 minutes.

### 2.6. Quality Control

The questionnaire was pretested to determine its appropriateness and suitability for the study. This resulted in corrections, rephrasing of some questions and rearrangement of sections of the questionnaire. Pretesting was done using 20 ANC attendants over a period of two days (10 per day) at the Atlantis hospital in the Kpone district with similar health services. To ensure uniformity of the process, the research assistants involved in the data collection were trained for five days on how to explain the study objectives, conduct the interviews, and obtain informed consent. Data extracted from the ANC cards were verified by a supervisor at the facility. Some of the data collected directly from the women were cross-checked from the ANC cards and registers and whenever there were discrepancies, what was on the ANC cards was used for this work.

### 2.7. Data Processing and Analysis

Data entry was done in Microsoft Excel software version 2013, cross-checked for completeness, and imported into Stata version 14 for cleaning and analysis. The data were summarized using descriptive statistics including frequencies, percentages, means, standard deviation, and ranges. The uptake of IPTp-SP was categorized into < three doses versus ≥ three doses. The sociodemographic and ANC characteristics were also grouped into categories. Chi-square/Fischer Exact tests were conducted to establish association between uptake of IPTp-SP and each independent categorical variable. Any association with a p-value < 0.05 was considered statistically significant. Logistic regression analysis reporting odds ratio was used to determine the strength of association between uptake of IPTp-SP and any significant independent variable that was found after the chi-square test.

Analysis of the qualitative data was done manually. The data were summarized using themes that brought similar views from different respondents together. Generally, variables were ranked high or good if six or more respondents provided similar answers and ranked low or poor if less than six of them provided similar answers.

## 3. Results

### 3.1. Background Characteristics of Study Participants

A total of 382 pregnant women in their eighth or ninth month of pregnancy (210, 55.0%) and nursing mothers who had delivered within the past six months (172, 45.0%), aged 18-45 years (mean: 29.3 years; SD: 5.4), participated in the study. Most of the women were married (77.8%), had basic level education, were self-employed (72.0%) and had one or two children (mean: 1.8; range: 0-7; SD: 1.2). Forty-one (3.7%) of the women, however, were without any child as at the time of data collection (Table 1).

### 3.2. Time of ANC Initiation and Number of Visits

**Figure 1:**
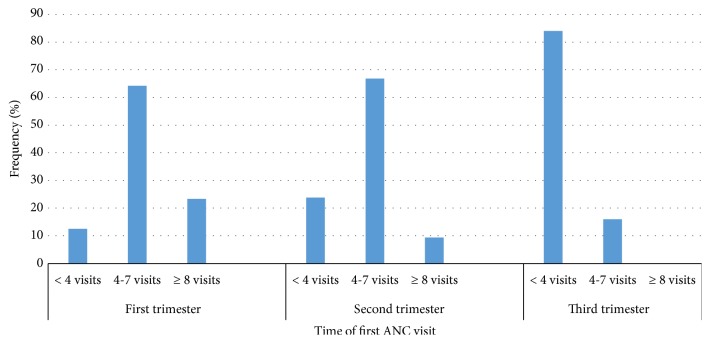
Time of first ANC visit and total number of visits made.

All the women attended ANC at least once during the most recent pregnancy. The average number of ANC visits was 5.3 (range: 1-10), 18 (4.7%) of the women made only one visit each whilst 58 (15.2%) made ≥ 8 visits each. One hundred and seventy-six (46.1%) of the women initiated ANC during the first trimester with most of them, 47.4% (181/382) initiating ANC during the second trimester. Only 25 (6.5%) of the women initiated ANC during the third trimester. Early initiation of ANC resulted in a higher number of visits. Out of the 176 women who made their first ANC visit during the first trimester, 41 (23.3%) were able to make ≥ 8 visits, whilst only 17 (9.4%) of those who initiated their visit during the second trimester were able to make ≥ 8 visits. None of those who were late (third trimester) in initiating ANC was able to make ≥ 8 visits (Figure 1). Thus the earlier ANC was initiated, the more likely it was for one to make more visits (*χ*^2^ = 74.67, p <0.001).

The number of ANC visits varied significantly between those who had delivered and those who were yet to deliver with 68.6% of those who had delivered making ≥ 4 visits compared to 57.1% of those who were yet to deliver (*χ*^2^ = 9.06. p = 0.003, Mentel-Haenszel corrected). No difference was however found between the ability of these two groups of women to make ≥ 8 visits (p >0.05).

### 3.3. Time of ANC Initiation and IPTp-SP Uptake

**Figure 2:**
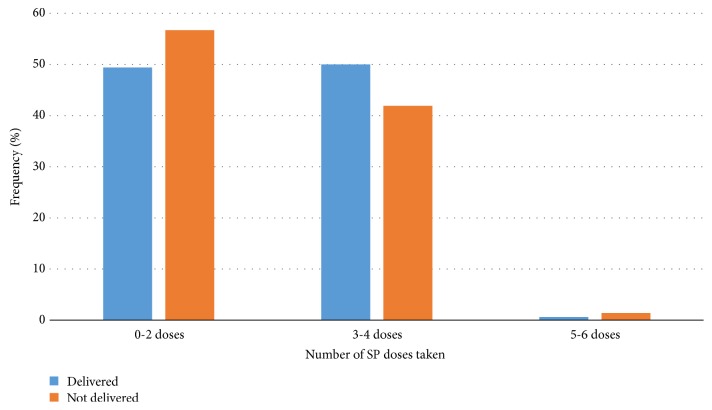
Doses of SP taken by mothers who had delivered and those who had not yet delivered.

Most of the women (98.2%, 375/382) took at least one dose of SP during the most recent pregnancy (mean: 2.3 doses; range: 0-6), with three (0.8%) and one (0.3%) of the women taking five and six doses respectively. Seven (1.8%) of the women did not take any dose of SP. Overall, 46.6 % (178/382) of the women took ≥ 3 doses of SP with only four (1.1%) taking ≥ 5 doses. Uptake of IPTp-SP was similar for women who had delivered and those who were yet to deliver (Figure 2) (*χ*^2^ = 2.94, p = 0.230).

**Figure 3:**
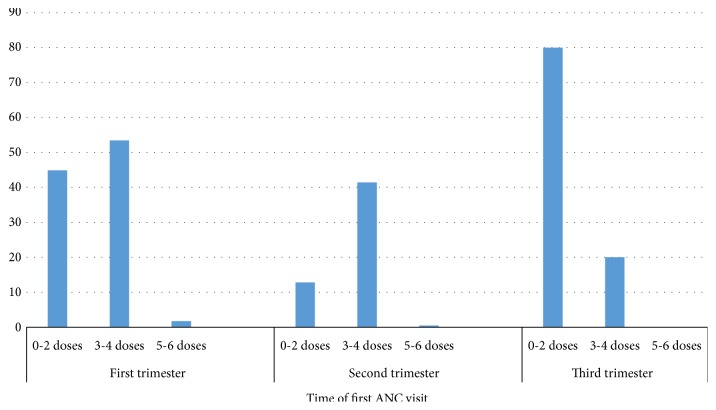
Time of first ANC visit and number of SP doses received.

Time of ANC initiation was found to be significantly associated with IPTp-SP uptake. The earlier the initiation, the higher the number of doses of SP taken (*χ*^2^ = 14.47, p = 0.006). Out of the 176 women who initiated ANC during the first trimester, 94 (53.4%) received 3-4 doses and three (1.7%) received 5-6 doses. Thus, 97 (55.1%) of the women who initiated ANC during the first trimester received ≥ 3 doses of SP whilst 42.0% (76/181) of those who initiated ANC during the second trimester received ≥ 3 doses of SP (*χ*^2^ = 5.64, p = 0.02). Similarly, those who initiated ANC during the second trimester received more SP doses compared to those who initiated the visit during the third trimester (*χ*^2^ = 4.43, p = 0.04) (Figure 3).

### 3.4. Individual and Service Related Factors and IPTp-SP Uptake

No association was found between individual factors investigated in this study and IPTp-SP uptake (Table 2). Several service related factors were also assessed to determine their level of influence on IPTp-SP uptake. The only factor found to be significantly associated with uptake of IPTp-SP was education/counselling given to the women on the significance of IPTp-SP by the attending midwives at the ANC. Among those who indicated that they were given prior education/counselling on SP, 31.9% took ≥ 3 doses compared to those who were not given any education/counselling on SP (13.6%) (p=0.001). Of the 382 women studied, 288 (75.4%) indicated that DOT was practiced at the facilities surveyed with 250 of them (65.4%) willing to take the drug under observation by the midwives. However, 111 (29.1%) indicated that they had never taken the drugs home before swallowing (Table 3).

Simple logistic regression was further used to assess the level of the associations between variables that showed significant effect on uptake of IPTp-SP. Results from the logistic regression indicated that respondents who had no information given to them by midwives prior to administration of SP had a lower odds of 2.2 of taking SP compared to those who had prior information (OR 2.2, 95% CI: 1.34-3.47). Also, respondents who had attended ANC > 5 times increased their uptake of IPTp-SP by 81% compared to those who had attended ≤ 5 times (OR 0.19, 95% C.I 0.12-0.31). Timing of first ANC visit was strongly associated with uptake. First ANC visit in the last trimester was seen to reduce the odds of uptake of at least three doses of SP by 6.2 compared to those who attended in the first trimester (Table 4).

### 3.5. In-Depth Interviews with Midwives on Facility Factors Likely to Affect IPTp-SP Uptake

In-depth interviews were held with twelve midwives in charge of the antenatal clinics to assess facility factors likely to affect uptake of IPTp-SP. These were made up of six staff midwives, two principal midwifery officers, and four retired midwives.

Overall, the level of knowledge on the standard guidelines on IPTp-SP was found to be low, whilst knowledge on the side effects of SP was high. Eleven of the twelve (91.7%) midwives interviewed could mention only two guidelines each out of the seven standard WHO guidelines on the administration of IPTp-SP. Those mentioned were starting SP after quickening and giving the drug at monthly intervals till delivery, as indicated in one of the quotations below.

“SP is taken during pregnancy at 16 weeks or after quickening”  (staff  midwife, Tema  East).

All twelve respondents, however, expressed good knowledge about the side effects of SP, including dizziness, weakness, headache, and vomiting as stated below.

“Some side effects of SP are dizziness, vomiting and weakness” (*principal*  *midwifery*  *officer*, *Tema*  *East*).

Knowledge on malaria in pregnancy and the need for IPTp-SP was also good. Ten out of the twelve (83.3%) respondents mentioned four main effects of malaria infection during pregnancy. They included spontaneous abortion, maternal anemia, intra uterine growth retardation, and low birth weight. However, two respondents were unable to mention any effect of malaria on pregnancy; the response of one of them to the question regarding malaria in pregnancy was that

“It is a long time I came on retirement, I do not remember those things”  (*retired*  *midwife*,   *Tema*  *Central*).

Respondents from seven of the twelve facilities said they educate the women on malaria at each ANC visit. The five remaining respondents said that even though health education is done at each ANC visit, malaria education is done occasionally since there are several other topics to be treated. According to one of them:

“We educate them at each ANC visit on how to protect themselves from mosquitoes. We even give them mosquito nets to use if available”  (midwifery officer, Tema East).

Probing further to inquire if detailed health education is given on IPTp-SP, ten out of the twelve (83.3%) midwives answered in the negative. According to them, education on IPTp-SP comes up only when the day's topic for health education is on malaria. The interview revealed that IPTp-SP is not strictly administered as DOT in the facilities surveyed. The midwives interviewed confirmed that DOT is not strictly enforced because the women have the preference of taking the drug home to swallow in the comfort of their homes and they are allowed because they are private facilities. In two of the facilities, the midwives indicated that the women collect the drugs from the pharmacy, and so the midwives do not have the opportunity to implement DOT or document uptake of the drug. According to one of the midwives:

“The pharmacy keeps all the SP drug so if a client is due for the drug, I write it on a prescription form for her to go to the pharmacy and collect”  (retired  midwife, Tema  Central).

This statement by the midwife supports the indication by 111 (29.1%) of the women that they have ever taken SP home before swallowing.

Findings in ten of the facilities showed effective documentation on IPTp-SP uptake. The midwives indicated that IPTp-SP uptake is documented on the antenatal cards as well as in the ANC register, which is the standard protocol for documentation of SP. Number of uptake of IPTp-SP for each client is documented against her name in the register.

### 3.6. Program Related Factors Likely to Affect IPTp-SP Uptake

In-depth interviews with the midwives on NMCP related factors likely to affect IPTp-SP uptake centered on the following themes: involvement in training programmes, supply of drugs and monitoring, and evaluation by the control programme. According to all the midwives, the NMCP organizes frequent workshops and always invited them to attend. One of the midwives indicated that* “Yes they invite us for programmes, I went for one in April” *(staff midwife, Tema East).

When asked about how timely and adequate the supply of SP, the common response was that supply was based on the request from the facility and that timely submission of request resulted in timely supply of drugs as indicated by a staff midwife, from Tema East.

“Yes we are supplied on timely basis but you see, supply depends on your request, if you send your request early, they will supply you”.

Regarding whether they had ever run out of supply and the effect it had on IPTp-SP administration, all the respondents said yes but that happened only once when the national drug store got burnt. A majority also added that they have never run out of stock after the fire incident since measures were put in place to solve the shortage problems. One of the extracts from the recordings was

“Just once when the national drug store got burnt, there was shortage of the drug but since they rectified it, we have never run out”  (retired  midwife, Tema  Central)

The interview revealed that the level of monitoring and evaluation by program implementers in the private facilities was good. Eight respondents reported that NMCP representatives from the submetropolitan unit come occasionally but four of midwives stated that the program implementers do not come on monitoring visits. However all twelve respondents stated that they submit monthly reports on IPTp-SP uptake to the metropolitan unit of the NMCP. According to a staff nurse from Tema West,* “report is submitted every month, if we delay and do not send it, they call us from the metro office”.*

## 4. Discussion

A cross-sectional survey was carried out among antenatal and postnatal mothers to investigate factors associated with uptake of IPTp-SP in private health facilities in the Tema metropolitan area of Ghana. The outcome of the survey revealed that early initiation of ANC resulted in a higher number of visits and uptake of SP, there was regular supply of SP and regular training workshops organized by the National Malaria Control Program (NMCP) for the facility staff but DOT was not strictly adhered to.

The average number of ANC visits of five recorded in the current study though higher than the number (four) recommended in the previous WHO policy on ANC visits [11] was much lower than the minimum recommended number of eight contacts in the new policy [12]. The proportion of women who made the recommended eight or more visits (15.2%) was, however, much higher than that observed in an earlier study in a public health facility in the capital city Accra [8]. More ANC visits are expected to create the opportunity for the attending midwife to identify pregnancy related problems including hypertensive disorders and have them treated to reduce maternal and infant morbidity and mortality [12].

Early initiation of ANC visits is essential as it can enable the woman make more visits and therefore increase the chances of benefiting from interventions put in place by the health authorities including uptake of IPTp-SP [13, 14]. A relatively high proportion of women (46 %) initiated ANC visits during the first trimester. This is much higher than reports from several other studies in Africa including, the 18-37% from Ethiopia [15–18], 32% from Nigeria [19], and 18% from Tanzania [20]; but the same as that reported by Muhwava and colleagues from South Africa [21]. This is, however, much lower than that reported (71%) by Sumankuuro and colleagues [22] for rural women in the Upper West region of Ghana.

ANC initiation during the first trimester has been associated with several factors including, being resident in an urban area [17], having higher education [19], being married, being employed, and the particular pregnancy being wanted [21]. Early initiation of ANC is crucial in being able to achieve the target of eight or more visits. In our current study, 23% of the women who initiated ANC during the first trimester were able to make the required ≥ 8 visits with only 9 % of those who initiated their visit during the second trimester being able to do so. Thus, the earlier ANC was initiated, the more likely it was for one to make the require number of visits before delivery.

As expected, more of the participants (69%) who had delivered were able to make ≥ 4 ANC visits compared to those who were yet to deliver (57.1%) as those yet to deliver still had some time to make more visits and possibly take more doses of SP as the new WHO policy requires. No statistically significant difference was however observed between the abilities of these two groups of women to make ≥ 8 visits. The ≥ 4 ANC visits recorded in our current study in these urban private health facilities was much lower than the 86% reported for rural communities in the country [23].

Making more ANC visits is known to be associated with taking more doses of SP [13, 14] and having better pregnancy outcomes [24]. Several individual level factors including the socioeconomic status of the woman [23], her employment status, her husband's educational attainment [25], community level factors [18], and health facility level factors including quality of ANC services provided [25] have all been associated with making ≥ 4 ANC visits.

Using a nationwide data source, Tenkorang [26] reported that women attending private health facilities in Ghana made more ANC visits than those using public health facilities. This observation was explained on the basis of users of private facilities being worthier and more educated than those using public health facilities. However, the overall uptake of ≥ 3 doses of SP of 47 % and the 51% by those who had delivered are much lower than the 89% uptake recorded for a public health facility in Accra [8]. Thus, women attending private health facilities may have reasons for attending ANC other than receiving more IPTp-SP doses. Such reasons may include quality of service [25], geographical access [27], and prompt attention leading to more client satisfaction. In the view of Tenkorang [26], however, ANC services in private health facilities in Ghana may not be better than that provided in public facilities; in this case, therefore, access could be a factor influencing the number of ANC visits [27].

None of the individual and facility level factors investigated in the current study established any association with ANC visits or uptake of IPTp-SP except for health education given by the midwives during ANC. Health education provided by attending midwives is important in ensuring that pregnant women attend ANC regularly and on time [16] as this enables the women to appreciate the seriousness of malaria in pregnancy [28]. The quality of interaction between the health care provider and the client is essential in IPTp-SP uptake [29] as pregnant women will usually accept IPTp-SP if encouraged by a health care provider to do so [30].

In-depth interviews with the midwives revealed that the supply of SP was not a problem [8] as supplies were readily available from the NMCP provided the request was made on time. Other studies have revealed that shortage of SP at the facility level can be a barrier to achieving high uptake of IPTp-SP [31–33]. Similarly, regular training for ANC service providers and supervision is important in improving uptake of SP [31]. Regular training on IPTp-SP guidelines and supervision opportunities for health workers results in improving their confidence and knowledge about the safety and efficacy of SP [30].

Directly observed treatment by attending midwives is an important component of the IPTp-SP policy which ensures that doses of SP given out to pregnant women are actually swallowed. Unfortunately, the level of compliance to this component of the guidelines was very low, as admitted by the midwives themselves during the in-depth interviews and supported by 29% of the women who indicated that they have ever taken the SP home. According to some of the midwives, because they operate private facilities, they are not able to impress upon the women to take the medication under observation. Low levels of compliance to the DOT policy have been reported for both private and public health facilities in earlier studies in Tanzania [28], Kenya, [34], and Nigeria [35, 36]. Such issues as availability of water and cups at the ANC units have been reported to serve as barriers to the effective implementation of DOT. Some private midwives also do not think it was necessary to practice DOT as they expect the women to take their drugs at home [36].

## 5. Conclusions

The current study in an urban private health facility in Ghana has established that early initiation of ANC resulted in a higher number of visits and uptake of SP. The facilities studied did not have problems with supply of SP and that staff of the facilities were regularly invited to participate in training workshops organized by the NMCP. The level of uptake of ≥ 3 doses of SP could be much higher than has been captured by the NMCP as in some of the facilities SP is dispensed through the pharmacy directly to the mothers and not through the midwives. In such cases, reports to the NMCP may not be complete. Uptake of SP could even be higher than we were able to capture in the current study as some of the women may receive some more doses of SP before delivery. The main problem identified that could adversely affect IPTp-SP uptake was the noncompliance with DOT by staff of the facilities. Further discussions with the NMCP could help develop ways of addressing this problem.

## 6. Limitations of the Study

The main limitations of the study include recall bias from nursing mothers who may not be able to recollect correctly all that happened during the ANC visits. For some of these mothers, it was not possible to verify the information which was self-reported because some of them did not carry their antenatal cards to the postnatal unit. Respondents who had not yet delivered still had the opportunity to take more doses of SP; this could result in some form of underrepresentation of uptake of ≥ 3 doses. These limitations notwithstanding this report give a fair idea of the level of uptake of IPTp-SP and factors influencing uptake. Addressing these factors could help improve the level uptake in private health facilities.

## Figures and Tables

**Table 1:** Sociodemographic characteristics of study participants.

Variable	Frequency	%
*Age group*		
18-24	73	19.1
25-31	179	46.9
32-38	107	28.0
39-45	23	6.0
*Marital status *		
Single	25	6.5
Married	297	77.8
Cohabiting	60	15.7
*Education *		
No formal education	60	15.7
Basic	198	52.0
Secondary	97	25.5
Tertiary	26	6.8
*Occupation *		
Government/Private	33	8.6
Self employed	275	72.0
Unemployed	74	19.4
*Number of children *		
0	41	10.7
1-2	243	63.6
3-4	87	22.8
5-7	11	2.9

**Table 2:** Sociodemographic characteristics of participants and IPTp-SP uptake.

*Variables *	*Frequency*	*IPTp-SP uptake (*%)			*p-value*
	N = 382	0-2 doses	3-4 doses	5-6 doses	
*Age group*					0.855
18-24	73	50.7	48.3	0.0	
25-31	179	54.7	43.6	1.7	
32-38	107	54.2	44.8	0.9	
39-45	23	47.8	52.2	0.0	
*Marital status*					0.383
Single	25	60.0	40.0	0.0	
Married	297	53.5	45.8	0.7	
Cohabiting	60	50.0	46.7	3.3	
*Education*					0.786
No formal education	60	51.7	48.3	0.0	
Basic	198	51.0	47.5	1.5	
Secondary	97	58.8	40.2	1.0	
Tertiary	26	57.7	42.3	0.0	
*Occupation*					0.727
Government/Private	33	48.5	51.5	0.0	
Self employed	275	51.5	45.1	1.5	
Unemployed	74	55.4	44.6	0.0	
*Number of children*					0.097
0	41	51.2	46.3	2.4	
1-2	243	51.4	48.1	0.4	
3-4	87	59.8	39.1	1.1	
5-7	11	54.5	36.4	9.1	

**Table 3:** Implementation of DOT, drug side effects, and IPTp-SP uptake.

*Variables *	*Frequency*	*IPTp-SP uptake (*%)			*p-value *
	N = 382	0-2 doses	3-4 doses	5-6 doses	
*Implementation of DOT*					0.109
DOT observed	288	39.8	34.6	1.0	
DOT not observed	87	11.8	11.0	0.0	
NA	7	1.8	0.0	0.0	
*Prior information on drug by nurse *					0.001
Information given	231	27.5	31.9	1.0	
No information given	144	24.1	13.6	0.0	
NA	7	1.8	0.0	0.0	
*Experience of drug side effects *					0.133
Experienced side effects	150	19.9	19.1	0.3	
Side effect not experienced	225	31.7	26.4	0.8	
NA	7	1.8	0.0	0.0	
*Preference of DOT*					0.132
Likes taking SP under DOT	250	35.3	29.3	0.8	
Does not like taking SP under DOT	125	16.2	16.2	0.3	
NA	7	1.8	0.0	0.0	
*Ever taken drugs home *					0.172
Ever taken	111	15.7	13.7	0.3	
Never taken	264	35.9	32.5	0.8	
NA	7	1.8	0.0	0.0	
*Malaria infection while on IPTp-SP*					0.122
Had malaria	41	6.3	4.5		
Did not have malaria	337	45.3	41.1	1.0	
NA	7	1.8	0.0	0.0	

**Table 4:** Crude and adjusted associations between variables and uptake of IPTp-SP.

Variable	Crude OR	95%CI	Adjusted OR	95%CI
*Drug information by nurses*				
Information given	0.46	0.30-0.70	2.16	1.34-3.47
No information given	1		1	
*Timing of 1st ANC visit*				
1st trimester	1		1	
2nd trimester	1.71	1.13-2.59	1.18	0.73-1.90
3rd trimester	6.20	2.04-18.89	2.18	0.65-7.31
*Number of ANC visits*				
≥ 5	1		1	
< 5	0.17	0.11-0.2	0.19	0.12-0.31

## Data Availability

All data generated during the current study are included in this published article and its supplementary information file (additional file 1).

## List of Abbreviations

ANC: Antenatal care
DOT: Directly observed treatment
GNMCP: Ghana National Malaria Control Programme
LLIN: Long-lasting insecticidal net
IPTp-SP: Intermittent preventive treatment of malaria in pregnancy with Sulfadoxine-Pyrimethamine
PMI: President's Malaria Initiative
PNC: Postnatal clinic
SP: Sulfadoxine-Pyrimethamine.

## Supplementary Materials

Supplementary Materials All data generated during the current study are included in this published article and its supplementary information file (additional file 1). Additional file 1:* Data set1.*
